# The Legality of Unqualified Practice

**Published:** 1898-07-16

**Authors:** 


### THE LEGALITY OF UNQUALIFIED PRACTICE.

The Collins case has brought markedly before the public the evils connected with the present state of the law as to the practice of medicine by persons who, although not qualified medical practitioners, yet assume titles which are commonly accepted by the laity as implying the possession of a legal qualification. Notwithstanding the fact that the defendant's name had been erased from the Register by the General Medical Council, he still retained his degree of M.D., and there can be no doubt that the use of this title led people who were unacquainted with the facts to believe that he was still a qualified medical man. It is a most unsatisfactory condition of affairs that an unqualified man should be able to publicly use a title which is commonly taken as indication expressing the possession of a legal qualification. It is still more unsatisfactory that no one should know exactly what the law on the subject is. It is maintained by some, notably by Mr. Horsley, that a man who practises without being on the Register violates the law, and we are pleased to note that last week the Deputy Coroner for East London definitely maintained that a man who was not a qualified medical practitioner, although he possessed a Munich M.D., rendered himself liable every time he visited or prescribed. In this case the coroner, in summing up, is reported to have said "he should feet it his duty to inform the proper authorities, so that this gentleman might be prosecuted. He had no English qualification and sailed under false colours, thereby deceiving the public." That is exactly the contention. Unfortunately that view does not appear as yet to have been maintained, or, indeed, to have been fully argued before the High Court, and magistrates have been found in plenty to decline to accept it. Nothing can be more improper than that the exact meaning of the law on a grave matter of this kind should remain a matter of doubt, and it is much to be hoped that the action of the coroner in the case we have just alluded to may lead to some pronouncement on the subject. The case seems a fairly typical one. The man boldly maintained that he was a medical practitioner, and that, although he had no power of recovering his fees, he had a right to practise. That is just the point we want deciding. It is not merely a matter of the right to practice, but of the right to use a title, however obtained, which misleads people as to a man's qualification. If he has no right the law is on our side, and it must be put in force against a multitude of offenders. If he has a right, it is absolutely essential for the protection of the public that the law should be altered at once.

